# TMT-Based Proteomics Reveal the Mechanism of Action of Amygdalin against Rheumatoid Arthritis in a Rat Model through Regulation of Complement and Coagulation Cascades

**DOI:** 10.3390/molecules28207126

**Published:** 2023-10-17

**Authors:** Lan Zhou, Jun-Hong Chai, Yi Zhang, Xiao-Jie Jing, Xiang-Wen Kong, Jun Liang, Yong-Gang Xia

**Affiliations:** Key Laboratory of Basic and Application Research of Beiyao, Heilongjiang University of Chinese Medicine, Ministry of Education, 24 Heping Road, Harbin 150040, China

**Keywords:** TMT-based proteomics, amygdalin, rheumatoid arthritis, complement and coagulation cascade

## Abstract

The limitations of current medications for treating rheumatoid arthritis (RA) emphasize the urgent need for the development of new drugs. This study aimed to investigate the potential anti-RA mechanism of amygdalin using tandem mass tag (TMT)-based quantitative proteomics technology. First, the anti-RA activity of amygdalin was evaluated in a Complete Freund’s adjuvant (CFA)-induced rat model. Then, the roles and importance of proteins in the extracted rat joint tissue were evaluated using TMT-based quantitative proteomics technology. A bioinformatics analysis was used to analyze differentially abundant proteins (DAPs). A proteomics analysis identified 297 DAPs in the amygdalin group compared with the model group, of which 53 upregulated proteins and 51 downregulated proteins showed opposite regulatory trends to the DAPs produced after modeling. According to enrichment analyses of the DAPs, the signaling pathways with a high correlation degree were determined to be the complement and coagulation cascades. Furthermore, western blotting and molecular docking were used to further validate the key node proteins, e.g., complement C1s subcomponent (C1s), component C3 (C3) and kininogen 1 (Kng1). These results suggest that amygdalin may be a promising agent for treating RA by regulating the complement and coagulation cascades.

## 1. Introduction

Rheumatoid arthritis (RA) is a chronic autoimmune disease that severely affects patients’ quality of life [1,2]. The main pathological features of this condition are inflammation and synovial hyperplasia, followed by the progressive destruction of articular cartilage and bone, which can ultimately lead to joint deformity and loss of function [3,4]. Currently, the main drugs used to treat RA are non-steroidal anti-inflammatory drugs, disease-modifying anti-rheumatic drugs, and glucocorticoid and biological agents [5]. However, they all have serious side effects (e.g., gastrointestinal damage, hepatorenal toxicity, cardiovascular risk) [6,7]. Currently, the limitations of common drug therapy have become some of the factors that severely affect the patients’ quality of life. Therefore, there is an urgent need to develop less toxic and more effective therapeutic drugs for RA.

The clinical application of traditional Chinese medicine in the treatment of RA has a long history, and modern pharmacological studies have also found that some Chinese herbs have good activity in the treatment of RA. Therefore, screening the effective ingredients of traditional Chinese medicine that are candidate drugs for the treatment of RA is a feasible approach.

**Figure 1:**
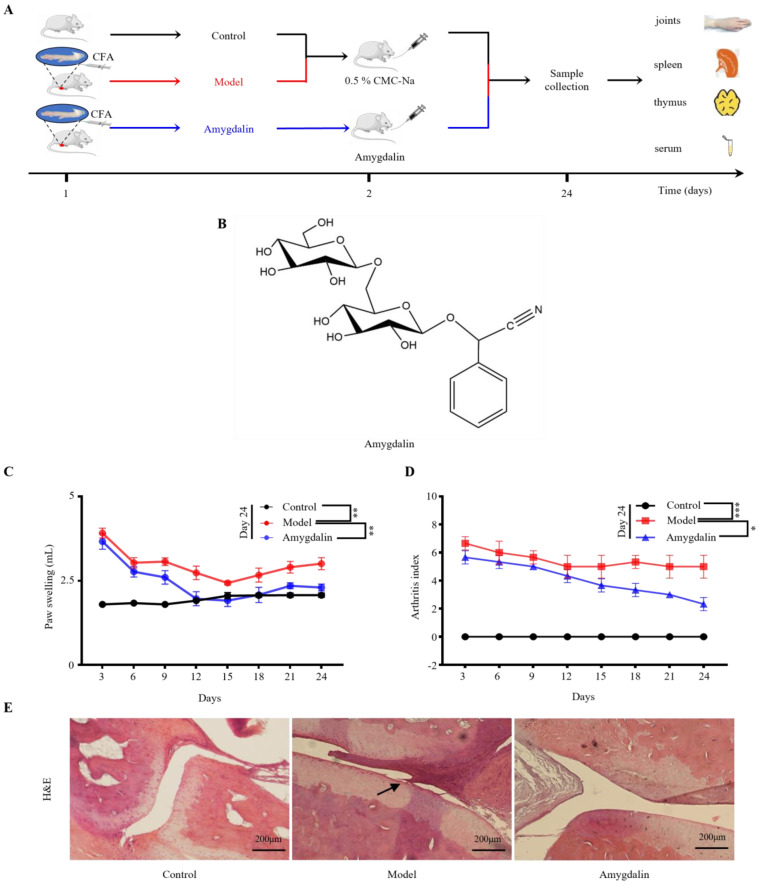
Effects of amygdalin on CFA-induced RA in rats. (**A**) Schematic diagram of the animal experimental design. (**B**) Chemical structure of amygdalin. (**C**) The degree of paw swelling in rats. (**D**) The arthritis index of CFA-induced arthritis rats. (**E**) Representative histopathological images of the articular structure stained with H&E. The black arrow indicates the location of the lesion. Control: normal control group; Model: CFA-induced model group; Amygdalin: amygdalin treatment for the CFA-induced group. Data are presented as the mean ± standard deviation (SD). * *p* < 0.05, ** *p* < 0.01, or *** *p* < 0.001 vs. model.

Amygdalin, which was first isolated by Robiquet and Boutron-Charlard in 1830 [8], is widely present in the seeds of Rosaceae plants such as peaches, plums, apples, and bayberry [9]. The molecular formula of amygdalin is C_20_H_27_O_11_N, and its structure is depicted in Figure 1B. In clinical practice, the Shentong-Zhuyu decoction (STZYD) has shown significant curative effects on RA [10], and amygdalin is a significant active component of Persicae Semen (Taoren), which is a key component in the STZYD. According to the Chinese Pharmacopoeia [11], amygdalin is a quality control indicator of Persicae Semen (Taoren), but its role in anti-RA treatment is unclear. Pharmacological studies have shown that amygdalin has many properties including anti-inflammatory, immunomodulatory, and analgesic effects [9]. Although amygdalin shows potential as an anti-RA drug, its specific mechanism needs further exploration.

This study first established a Complete Freund’s adjuvant (CFA)-induced rat model to explore the therapeutic effects of amygdalin on RA and its molecular mechanisms. Next, the anti-RA effects of amygdalin were evaluated by histopathology and cytokine detection. Then, tandem mass tag (TMT)-based quantitative proteomics was used to identify the differentially abundant proteins (DAPs). Bioinformatics analyses of the DAPs were performed to identify key proteins, which were then verified by western blotting. In addition, molecular docking was performed to analyze the interaction between amygdalin and these key proteins.

## 2. Results

### 2.1. Effects of Amygdalin on CFA-Induced Rats

#### 2.1.1. Effects of Amygdalin on Histopathology in CFA-Induced Rats

As shown in Figure 1A, CFA was injected subcutaneously into the bottom of the right hind paw on day 1 to establish CFA-induced rats. After successful modeling, rats in the treatment group were administered amygdalin (Figure 1B) by continuous intragastric administration for 22 days, after which the thymus, spleen, serum, and joints were collected.

As shown in Figure 1C, the degree of paw swelling of the right hind paw of the model group was significantly greater than that of the control group (*p* < 0.01). However, after treatment with amygdalin, the rats’ paw swelling gradually decreased. By day 24, the degree of paw swelling in the amygdalin group was markedly reduced compared with the model group (*p* < 0.01). Moreover, in rats treated with amygdalin, the arthritis index scores decreased from day 6, and by day 24, the scores of the amygdalin group were significantly lower than those of the model group (Figure 1D; *p* < 0.05).

The histopathological changes in rat articular structures were assessed and are shown in Figure 1E. The control group did not exhibit any damage, whereas the model group showed tissue adhesions. Following amygdalin treatment, hematoxylin and eosin (H&E) staining showed a significant improvement compared with the model group. It can be observed that the joint cavity structure is clear and the cartilage is orderly in the amygdalin group.

**Figure 2:**
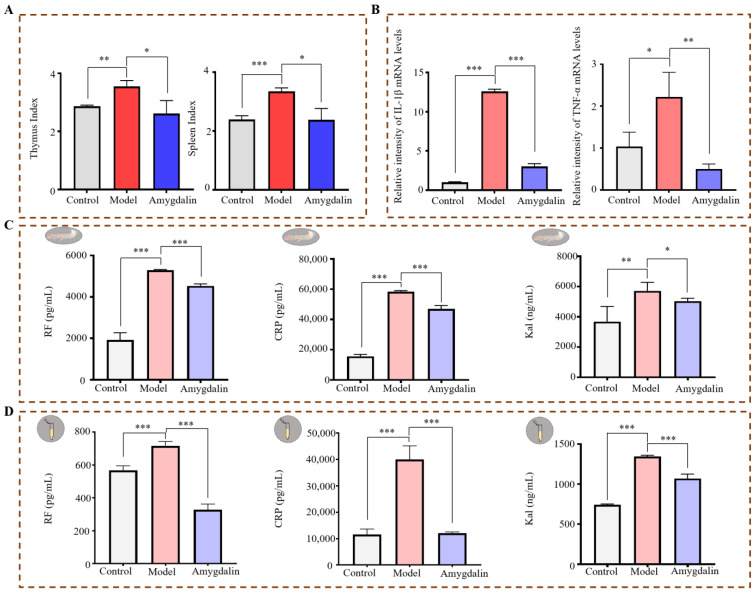
Organ index and the levels of inflammatory cytokines. (**A**) Organ index of spleen and thymus. (**B**) The mRNA levels of IL-1β and TNF-α in joints of rats (**C**) The levels of RF, CRP, and Kal in the joints of rats were detected with an enzyme-linked immunosorbent assay (ELISA) kit. (**D**) The levels of RF, CRP, and Kal in the serum of rats were detected with an ELISA kit. Data are presented as the mean ± SD (n = 3). * *p* < 0.05, ** *p* < 0.01, or *** *p* < 0.001.

As shown in Figure 2A, after sacrificing the rats, their spleen and thymus were weighed to calculate their organ indexes. There was a significant increase in the organ index of thymus and spleen in the model group (*p* < 0.01). The spleen and thymus indexes were significantly decreased in the amygdalin-treated groups compared with the model group (*p* < 0.05). This demonstrates that amygdalin reversed the immune dysfunction induced by CFA.

#### 2.1.2. Effects of Amygdalin on CFA-Induced Cytokines in Rats

The mRNA expression levels of interleukin 1 beta (IL-1β) and tumor necrosis factor alpha (TNF-α) in the joint tissues of rats in the three groups were detected. As shown in Figure 2B, the successful establishment of the CFA-induced rat model was indicated by significantly higher mRNA levels of TNF-α and IL-1β in the model group than in the control group (*p* < 0.05). Amygdalin treatment resulted in the decreased production of these pro-inflammatory factors (*p* < 0.01), demonstrating the excellent anti-inflammatory effects of amygdalin.

In the model group, the protein levels of rheumatoid factor (RF), C-reactive protein (CRP), and kallikrein (Kal) were significantly higher in the joints than those in the control group (*p* < 0.01), but those levels were significantly reduced after treatment with amygdalin (*p* < 0.05) (Figure 2C). The serum levels of RF, CRP, and Kal were significantly increased in the model group (*p* < 0.001) and were significantly decreased by amygdalin treatment (*p* < 0.001) (Figure 2D).

### 2.2. Identification of DAPs

**Figure 3:**
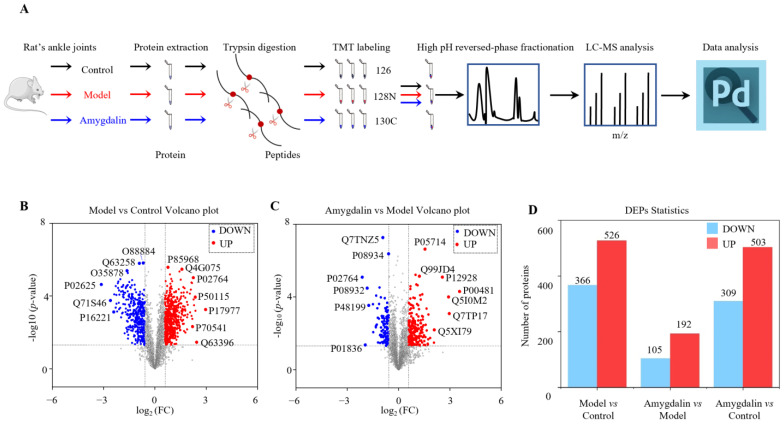
TMT-based proteomic study workflow and evaluation of upregulated and downregulated proteins. (**A**) Workflow of TMT-based quantitative proteomics study. (**B**) Volcano plot of DAPs in the model group compared with the control group. (**C**) Volcano plot of DAPs in the amygdalin group compared with the model group. Blue dots represent downregulated proteins, red dots represent upregulated proteins, and gray dots represent proteins with no statistically significant difference, based on fold change > 1.5 or <1/1.5 and *p* < 0.05. (**D**) Upregulated and downregulated proteins for three groups, i.e., model vs. control, amygdalin vs. model, and amygdalin vs. control.

To determine the mechanisms underlying amygdalin’s therapeutic effects on adjuvant arthritis in rats, TMT-based quantitative proteomics was used to evaluate the DAPs between the amygdalin and model groups (Figure 3A). A total of 4523 proteins with high reliability were screened. The volcano map of DAPs was drawn with the log_2_ (fold change) on the x-axis and −log_10_ (*p*-value) on the y-axis (Figure 3B,C). Based on the cutoff value of a 1.5-fold change, compared with the control group, 366 downregulated and 526 upregulated proteins were identified in the model group. Compared with the model group, 105 downregulated and 192 upregulated proteins were identified in the amygdalin group. Compared with the control group, 309 downregulated and 503 upregulated proteins were identified in the amygdalin group. (Figure 3D).

### 2.3. Gene Ontology (GO) and Kyoto Encyclopedia of Genes and Genomes (KEGG) Analyses of the DAPs

**Figure 4:**
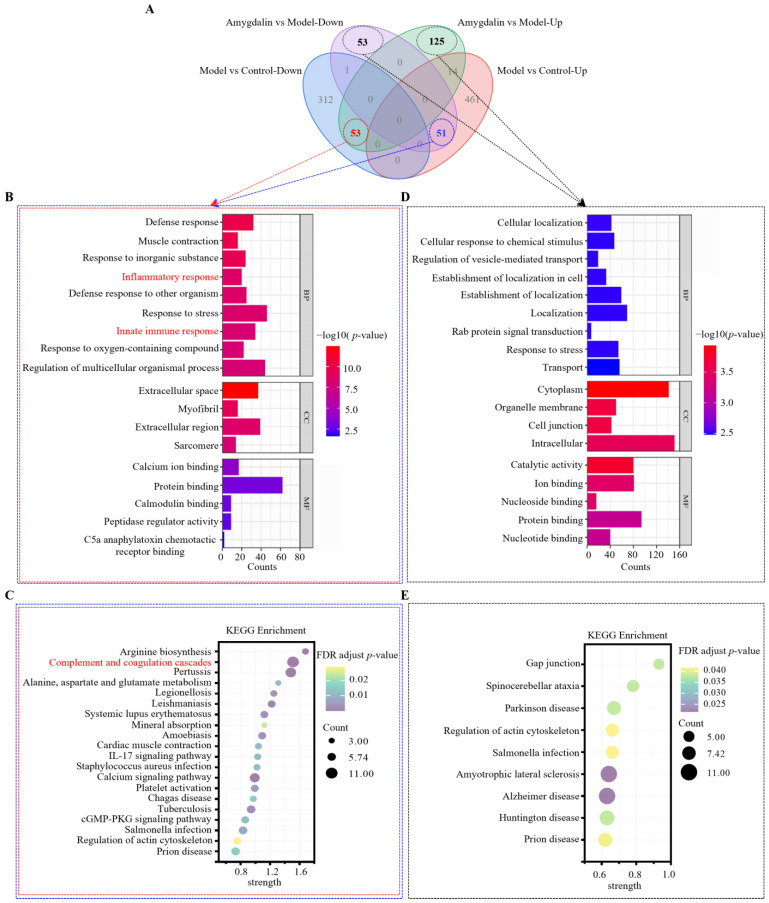
Enrichment analyses of DAPs. (**A**) Venn diagram of DAPs. The pink circle represents upregulated proteins in the model group compared with the control group, the blue circle represents downregulated proteins in the model group compared with the control group, the purple circle represents downregulated proteins in the amygdalin group compared with the model group, and the green circle represents upregulated proteins in the amygdalin group compared with the model group. Overlapping sections indicate the common proteins across different groups. (**B**) GO analyses of 53 plus 51 reversed DAPs (red and blue dashed lines) in the amygdalin group. (**C**) KEGG pathway enrichment analyses of 53 plus 51 (red and blue dashed lines) reversed DAPs (**D**) GO analyses 125 plus 53 non-overlapping DAPs (black dashed line) in the amygdalin group. (**E**) KEGG pathway enrichment analyses of 125 plus 53 non-overlapping DAPs (black dashed line) in the amygdalin group.

Venn diagrams were made for the upregulated DAPs and downregulated DAPs of the two groups (Figure 4A). The DAPs of the amygdalin group were further divided into two parts for enrichment analyses. Fifty-one proteins were upregulated in the model group but downregulated in the amygdalin group, and fifty-three proteins were downregulated in the model group but upregulated in the amygdalin group. The enrichment GO item bar graph was drawn according to the −log_10_ (*p*-value) of the enrichment analyses, from large to small (Figure 4B). GO enrichment analyses of the 51 plus 53 DAPs revealed their involvement in biological processes such as muscle contraction, inflammatory responses, and innate immune responses. Regarding the cellular component, the DAPs were localized in the extracellular space, myofibrils, extracellular region, and sarcomere. Regarding molecular function, the data suggested that the DAPs were significantly related to calcium ion binding, protein binding, calmodulin binding, peptidase regulator activity, and complement component 5a (C5a) anaphylatoxin chemotactic receptor binding. KEGG analyses showed that 51 plus 53 DAPs mapped to 20 KEGG pathways (Figure 4C). The main pathways comprised the complement and coagulation cascade and IL-17 signaling pathway. Among them, the complement and coagulation cascades were the pathways with the largest number of enriched proteins and the smallest *p*-value. In addition, the enrichment strength of this pathway ranked second overall. In summary, the complement and coagulation cascades were the signal pathways with the highest correlation degree.

As shown in Figure 4A,D, of the non-overlapping DAPs in the amygdalin group, 125 proteins are upregulated and 53 proteins are down-regulated. Enrichment analyses of 125 plus 53 DAPs were performed. GO enrichment analyses of 125 plus 53 DAPs were also carried out (Figure 4D). The results showed that the DAPs were significantly related to biological processes such as cellular localization, response to stress, and localization. In terms of the cellular component, the DAPs were primarily localized in the cytoplasm, and intracellular component. Regarding the molecular function, the DAPs were primarily involved in catalytic activity and ion binding. KEGG analyses showed that there were 125 plus 53 DAPs mapped to nine KEGG pathways (Figure 4E). The main pathways were involved in gap junctions, Parkinson’s disease, and regulation of the actin cytoskeleton.

### 2.4. Heatmap of the DAPs Related to Immune and Inflammation Pathways

**Figure 5:**
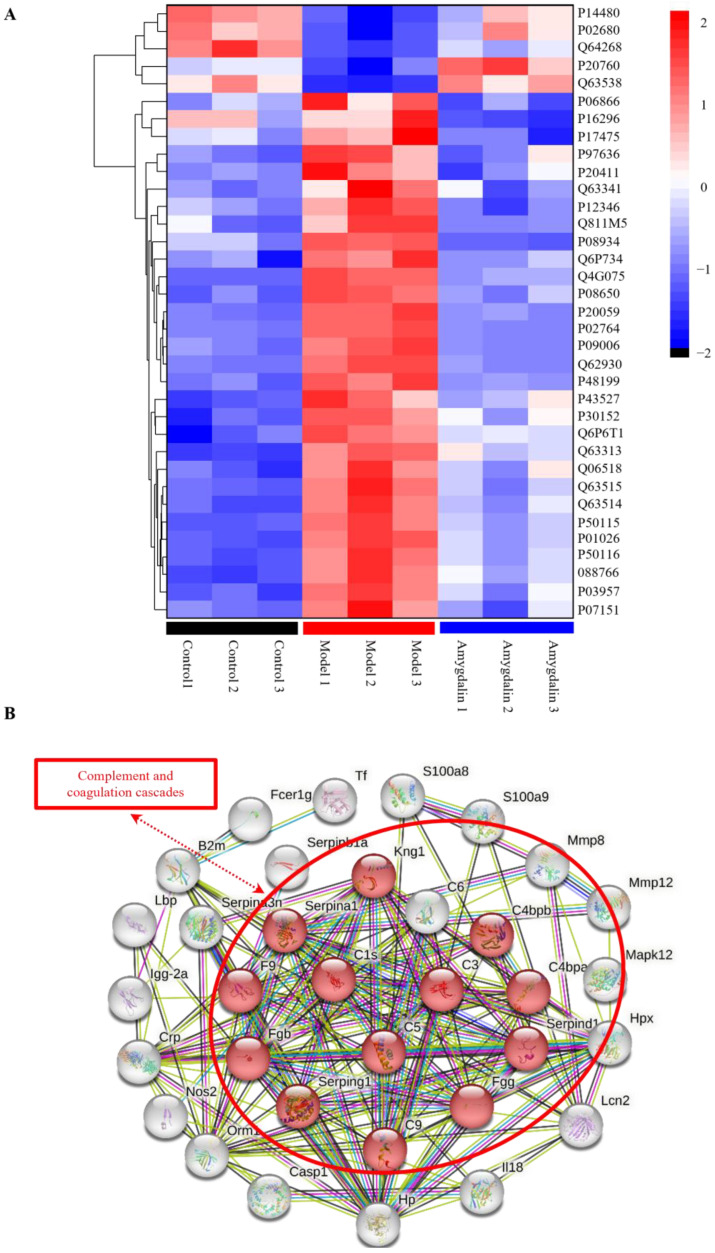
Clustering thermogram analyses and PPI analyses. (**A**) Heatmap of DAPs related to immune and inflammation pathways in the amygdalin versus model group. The differential expression of proteins is represented using a pseudo-color scale, with high levels of expression shown in red and low levels shown in blue. (**B**) PPI network diagram. Each node in the interaction network represents a protein and the connections between nodes indicate protein interactions.

RA is a chronic autoimmune disease mainly characterized by inflammatory synovitis, so cluster analyses were performed on all of the proteins enriched in GO and KEGG that were related to inflammation and immune function (Figure 5A and Table S1). The results showed that compared with the model group, most of the protein expression levels were reversed after amygdalin treatment.

As shown in Table S1, the fold change values of some proteins, such as complement C3 (C3), complement C1s subcomponent (C1s), and complement C5 (C5), were more than 1.5 after modeling and administration.

### 2.5. Protein–Protein Interaction (PPI) Analyses and Verification of Proteomics

To better understand the molecular mechanism of crosstalk among DAPs in the heatmap, we used the STRING database to examine their relationships. According to STRING analyses, a total of 34 nodes and 143 edges were interconnected (Figure 5B). The proteins in the complement and coagulation cascades showed high connectivity of other inflammation and immune-related proteins in the PPI network.

Some of these nodes have high confidence with an interaction score greater than 0.7, such as C3, C1s, C5, and kininogen 1 (Kng1).

### 2.6. Verification of Proteomics and Molecular Docking

**Figure 6:**
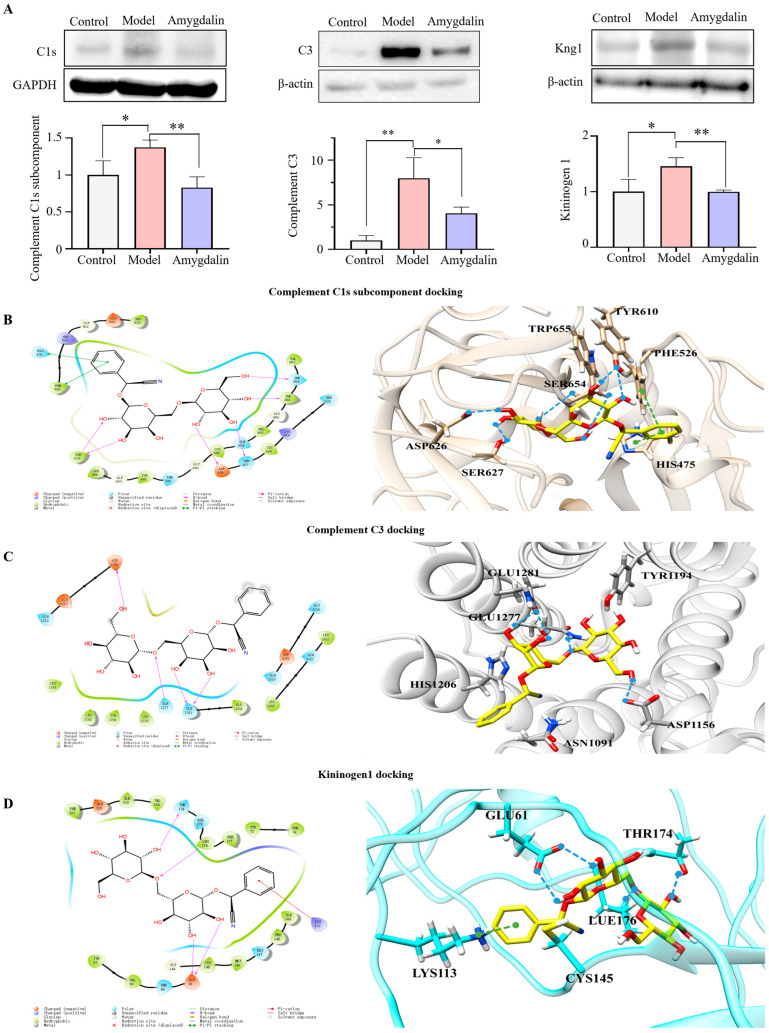
Verification of proteomics. (**A**) Western blot verification of C1s, C3, and Kng1. (**B**) Interactions of the C1s–amygdalin complex. (**C**) Interactions of the C3–amygdalin complex. (**D**) Interactions of the Kng1–amygdalin complex. The dashed blue lines represent hydrogen bonds and the green lines represent π-anion interactions. Data are presented as the mean ± SD (n = 3). * *p* < 0.05, or ** *p* < 0.01.

Among the differential proteins in the amygdalin group, the most obvious pathways were the complement and coagulation cascades; its targets included C3, C1s, and Kng1. We further verified these proteins by western blot analysis, which showed that the levels of C3, C1s, and Kng1 were significantly decreased after administrating the amygdalin when compared with the model group (*p* < 0.05; Figure 6A). The western blot results were consistent with the findings of the TMT proteomics analyses.

The docking results for the three targets, C1s, C3, and Kng1, are presented herein. Upon binding to C1s, it was observed that amygdalin’s multiple hydroxyl groups were oriented towards hydrophilic residues in the binding cavity, such as TYR610, TRP655, and SER627 with strong hydrogen-bonding interactions, possessing GlideScore as −7.285 kcal·mol ^−1^ (Figure 6B). The GlideScore is an empirical scoring function that approximates the ligand binding free energy. Furthermore, the benzene ring of amygdalin displayed obvious π–π hydrophobic interactions with HIS475 and PHE526. Upon binding to C3, multiple hydrogen bonds were observed between amygdalin and residues, such as ASP1156 and GLN1127 within the C3 binding cavity, showing GlideScore as −6.187 kcal·mol^−1^ (Figure 6C). Despite Kng1 being the result of homology modeling, it nonetheless demonstrated hydrogen bond interaction with GLU61 and LEU176, as well as π-cation hydrophobic interactions with LYS183, displaying GlideScore as −7.181 kcal·mol^−1^ (Figure 6D). The docking score further confirmed that amygdalin exhibited good binding affinity for each of these proteins.

**Figure 7:**
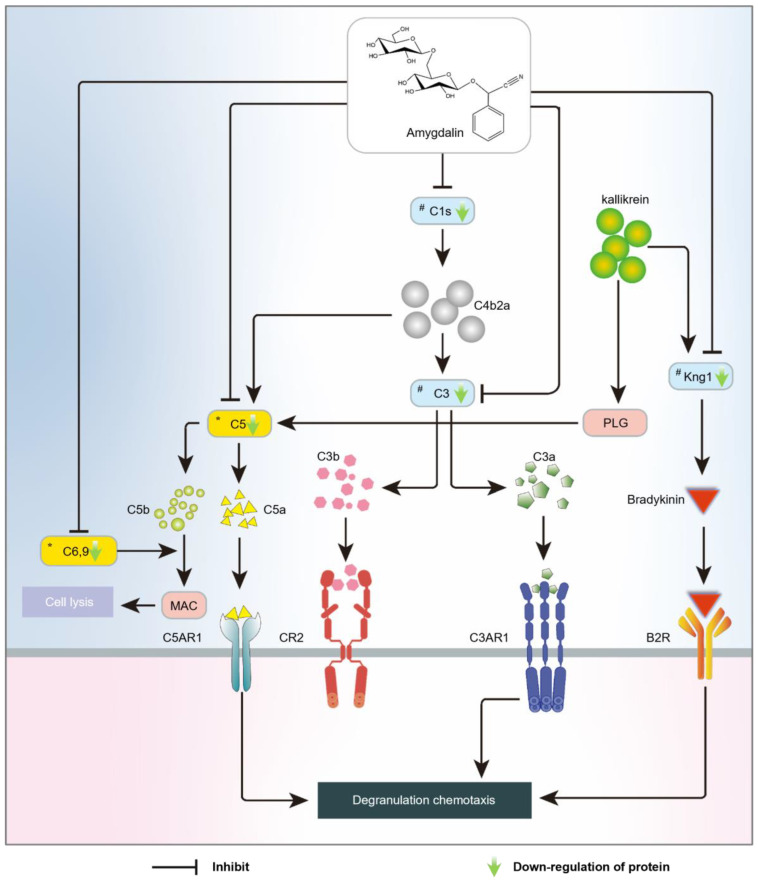
A hypothesized action mechanism of amygdalin against rheumatoid arthritis was proposed to be ascribed to the complement and coagulation cascades obtained from TMT-proteomics. Note: * DAPs found in TMT-based proteomics; # DAPs verified by western blot assays.

Overall, the above results show that the expression levels of C1s, C3, and Kng1 were significantly downregulated after amygdalin intervention. Additionally, since there are close relationships between DAPs (C1s, C3, C5, C6, C9 and Kng1) and their receptors (C5AR1, C3AR1, CR2 and B2R) [12,13], an action mechanism of amygdalin on rheumatoid arthritis was tentatively proposed in Figure 7. These proteins played an important role in the complement and coagulation cascade, which is closely related to inflammation and immunity.

## 3. Discussion

In this study, an initial CFA-induced rat model was established to evaluate the protective effects of amygdalin on RA. Then TMT quantitative proteomics technology was used to analyze the proteins of joint tissues of different experimental groups, with a focus on proteomic changes induced by CFA and the effects of amygdalin treatment.

The results from in vivo experiments showed that the thymus and spleen indices of CFA-induced model rats were significantly increased. Treatment with amygdalin significantly decreased these indices, demonstrating its immunomodulatory effects. TNF-α and IL-1β are pro-inflammatory cytokines involved in RA [14,15,16]. Thus, TNF and IL-1β receptor inhibition are considered novel targeted therapeutic strategies for RA [17,18]. CRP is an acute phase protein and a non-specific marker of inflammation and tissue damage [19,20]. In this study, several cytokines related to RA were significantly downregulated after intervention with amygdalin, suggesting that amygdalin may have anti-RA effects.

The proteomics results showed that amygdalin partially reversed the expression levels of DAPs in the model group. Enrichment analyses of these DAPs revealed their close association with immune regulation and inflammatory responses, whereas the other DAPs generated by amygdalin intervention had a low correlation with immune regulation and inflammatory responses. Further analyses of the DAPs reversed by amygdalin suggest that it may exert anti-RA effects by regulating the complement and coagulation cascades.

The importance of the complement system in innate immunity is evident, as it is involved in many pathological processes of autoimmune and inflammatory diseases, including RA [21,22]. The activation of complement is closely related to the onset of RA, the progression of arthritis, and the injury of the joint tissue [23,24]. The abnormal activation of complement leads to the damage of cartilage and bone structure, as well as the infiltration of synovium by a large number of T cells, B cells, macrophages, and granulocytes, making it an important factor in the pathogenesis of RA [25].

The complement system can be activated by three main pathways: classical, alternative, and lectin pathways [26,27]. C3 is the central molecule for complement activation [28], and its cleavage product C3a is a key effector molecule in the complement system, playing a role in both the classical and alternative pathways [29]. C3 is a key protein that is upregulated after CFA induction and downregulated after amygdalin intervention, and amygdalin has a good binding affinity for C3. These results suggest that amygdalin may inhibit C3 to exert anti-RA effects. C1s triggers the classical complement pathway, leading to the formation of C3 convertase, which further cleaves C3 into C3a and C3b [12]. In our experimental results, the expression of C1s increased in the model group, and decreased after treatment with amygdalin. This suggests that amygdalin may inhibit the formation of the C1 complex by binding to C1s. As a result, it suppresses the activation of the complement system’s classical pathway and reduces the production of the potent pro-inflammatory factor C3a. In addition, amygdalin can combine with C1s to form strong hydrogen bond interactions; as shown in Figure 6B, the result supports our conjecture. At the same time, this is consistent with reports in the literature that C1s inhibitors with similar binding modes were found to inhibit the activity of C1s [30]. These results suggest that on the one hand, amygdalin may indirectly inhibit the C3a cleavage process by suppressing the expression of the upstream molecule C1s in the complement system, and on the other hand, amygdalin may directly inhibit C3 to exert anti-RA effects.

As can be seen in Figure 7, C5, C6, and C9 can assemble into a membrane attack complex (MAC) [31], which is the final step of the C5 cleavage pathway in the complement system [32]. The MAC has the ability to lyse cells, leading to cell necrosis and apoptosis, or the release of inflammatory signals such as TNF-α and IL-1β [24], which further promote the inflammatory response in the joints. Meanwhile, C5a generated by the decomposition of C5 is also a powerful pro-inflammatory factor in the complement system [33]. Our experimental results suggest that amygdalin reduces the formation of MAC and C5a by downregulating C5, C6, and C9. It has been shown in previous studies that reducing the severity of arthritis by inhibiting the expression of C5 is possible [34]. Therefore, this may be one of the ways in which amygdalin alleviates the inflammatory response in RA model rats.

The kallikrein–kinin system is part of the coagulation cascade. As an important component of this system, Kal can cleave C3 and C5 into C3a, C3b, and C5a, and trigger the amplification loop [35]. This suggests a close relationship between the complement system and the clotting cascade. In addition, the activation of Kal induces the cleavage of Kng1, releasing bradykinin, which further promotes the inflammatory response [36,37]. Studies have shown that joint inflammation in mice lacking Kng1 was improved histologically, and the expression of inflammatory factors IL-1β and IL-6 was reduced [38]. The proteomic results indicated that amygdalin can decrease the expression of Kng1. In addition, the Enzyme-linked immunosorbent assay (ELISA) results also showed that amygdalin can inhibit the expression of Kal. In summary, it suggested that amygdalin may exert anti-RA effects by simultaneously inhibiting the expression of Kal and Kng1.

## 4. Materials and Methods

### 4.1. Experimental Animals

Specific pathogen-free-grade male SD rats (230 ± 20 g) were purchased from Liaoning Changsheng Biotechnology Co., LTD. (License no. SCXK (Liao) 20200001) (Changchun, China). Before the experiment, the animals were adaptively fed for one week under standard conditions. They were housed in a feeding room maintained at a temperature of 23 ± 2 °C and a humidity of 45 ± 5%, with good ventilation and dry conditions. The rats had access to adequate food and water. All animal experiments were approved by the Experimental Animal Ethics Committee of Heilongjiang University of Chinese Medicine and conformed to NIH guidelines (NIH Publications No. 85–23, revised in 1985).

### 4.2. CFA-Induced Rat Model Preparation and Treatment

The CFA-induced rat model is frequently utilized in RA. This model exhibits similar symptoms to human RA, including lymphocyte infiltration into the synovium, increased paw volume, cartilage destruction, and bone erosion [39]. To establish CFA-induced rats, 0.05 mL of CFA, containing 10 mg of lyophilized *Mycobacterium butyricum* in 1 mL of liquid paraffin [40], was subcutaneously injected into the bottom of the right hind paw on day 1. The animals were randomly divided into three groups (n = 10 per group): (1) The control group comprised normal rats that were orally administered a 0.5% Carboxymethyl cellulose sodium solution (CMC-Na). (2) The model group comprised CFA-induced rats that were orally administered a 0.5% CMC-Na solution. (3) The amygdalin group comprised CFA-induced rats that were orally administered 10 mg/g amygdalin in a 0.5% CMC-Na solution.

### 4.3. Measurement of Body Weight, Degree of Paw Swelling, and Arthritis Index Scores

Throughout the study, the body weights and right hind paw volumes of the rats were measured every three days. The arthritis score index was also recorded. The severity of arthritis in each paw was graded on a scale of 0–4:0, no swelling; 1, swelling of finger joints; 2, swelling of phalanx joint and digits; 3, severe swelling of the entire paws; 4, deformity, unable to bear weight. The maximum combined score should not exceed 16 points [41,42]. On day 24, the rats were sacrificed after weighing, and spleen, thymus, blood samples and joints were taken. The spleen and thymus of the rat were weighed, and the organ weight (mg) was divided by the body weight (g) of the rat to obtain the immune organ index.

### 4.4. Histopathological Observation

The right hind paws of rats were skinned and fixed in formalin solution for 8 h. The specimens underwent histopathology, and tissue samples were decalcified in 10% EDTA at 4 °C for 30 days. The tissues were dehydrated, processed, and embedded in paraffin wax after decalcification. The resulting blocks were then cut into 5 µm-thick sections, which were stained with H&E, and observed under a light microscope.

### 4.5. ELISA and Quantitative Polymerase Chain Reaction (qPCR) Analysis

The collected blood samples were centrifuged at 3000× *g* for 10 min to obtain serum. The levels of cytokines RF (Jianglai, Catalog number: JL21136, Shanghai, China), CRP (Jianglai, Catalog number: JL10287, Shanghai, China), and Kal (Jianglai, Catalog number: JL53075, Shanghai, China) in serum and joints of rats were detected by ELISA kit according to manufacturer’s instructions.

The mRNA levels of interleukin 1 beta (IL-1β) and tumor necrosis factor alpha (TNF-α) in joints of rats were detected. Total RNA was extracted using an RNA extraction Kit (Vazyme, Catalog number:RC112–01, Nanjing, China). RNA quality and quantity were determined using a spectrophotometer (Thermo Nano Drop™ 8000, Wilmington, DE, USA). Complementary DNA was synthesized from RNA (1 µg), using Reverse Transcription Master Mix (ABclonal, Catalog number: RK20429, Wuhan, China). qPCR was performed using SYBR Green PCR Master Mix (ABclonal, Catalog number: RK21203, Wuhan, China) on a CFX-384 system. qPCR primers are listed in Table 1.

### 4.6. TMT-Labeled Quantitative Proteomics

#### 4.6.1. Peptide Preparation

The frozen rat joint samples were ground into a powder with liquid nitrogen and lysed with RIPA buffer (Beyotime, Catalog number: P0013, Shanghai, China) containing a protease inhibitor mixture on ice for 30 min. The mixture was then centrifuged at 17,000× *g* at 4 °C for 25 min, and the supernatant protein concentration was determined with a BCA kit (Beyotime, Catalog number: P0012, Shanghai, China) according to the manufacturer’s instructions. 200 µg of protein from each sample were isolated using a 10 KD ultrafiltration tube and subsequently diluted with 8M Urea (UA)-100 mM Tris-HCl buffer. The sample was centrifuged at 12,000× *g* at 4 °C for 30 min, followed by alkylation with iodoacetamide in the dark at room temperature for 30 min. After centrifugation at 12,000× *g* at 4 °C for 15 min, the sample was substituted with 8M UA and 100 mM Triethylammonium Bicarbonate (TEAB) (Thermo Scientific, Catalog number: 90114, Waltham, MA, USA) buffer three times and then with 100 mM TEAB three times. Trypsin (Thermo Scientific, Catalog number: 90057, Waltham, MA, USA) digestion was performed overnight with a protease-to-protein ratio of 1:50. The mixture was centrifuged using a 10 KD ultrafiltration tube. The filtrate was collected.

#### 4.6.2. TMT Labeling

To quantify the peptides, the peptide quantification kit (Thermo Scientific, Catalog number: 23275, Waltham, MA, USA) was used. Next, 50 µg of peptides were extracted from each sample and labeled using a TMT pro10-plex Label Reagent Set (Thermo Scientific, Catalog number: 90110, Waltham, MA, USA) following the manufacturer’s protocol. Three rats in the control group were labeled with TMT-126, while three rats in the model group were labeled with TMT-128N, and three rats in the amygdalin group were labeled with TMT-130C. The labeling reagent was dissolved in acetonitrile and incubated with peptides for 1 h. The incubation was terminated with 50% hydroxylamine (Thermo Scientific, Catalog number: 90115, Waltham, MA, USA). The labeled peptides were then pooled, desalted using peptide desalting spin columns (Thermo Scientific, Catalog number: 89852, Waltham, MA, USA), and freeze-dried under vacuum.

#### 4.6.3. High pH Reversed-Phase Fractionation

The TMT-labeled peptides were fractionated using high pH reverse-phase HPLC on an Agilent 1260 Series HPLC Value system with a C18 column (Waters, Catalog number: 186003581, Milford, MA, USA). The mobile phase A was composed of 98% water and 2% acetonitrile (pH = 10), while mobile phase B was composed of 98% acetonitrile and 2% water (pH = 10). The pH value of the mobile phase was adjusted using an ammonium hydroxide solution. The column temperature was set to 50 °C, with a flow rate of 0.8 mL/min. The desalted peptide segment was dissolved in a solution of 95% A and 5% B. After centrifugation at 4 °C and 12,000× *g* for 15 min, the supernatant was collected. The peptide separation took 68 min using a gradient of 8–34% B. Using an automatic collector, one fraction was collected every two minutes. The fractions were then combined into 11 fractions and freeze-dried under vacuum for further processing.

#### 4.6.4. Nano LC-MS/MS Analysis and Proteomics Data Processing

The combined components were separated using the UltiMate 3000 nL flow liquid phase system. The chromatographic column (Thermo Scientific, Catalog number: 164940, Waltham, MA, USA) had particles of 2 μm, an inner diameter of 75 μm, and a length of 15 cm. The mobile phase A was composed of 0.1% formic acid—aqueous solution, while mobile phase B was composed of 80% acetonitrile (containing 0.1% formic acid). The column temperature was set to 55 °C, with a flow rate of 300 nL/min. Peptides tagged with TMT were dissolved in mobile phase A, separated using a 120-min gradient of mobile phase B (ranging from 3% to 35%). Samples were separated by an analytical column and analyzed by Orbitrap Fusion Lumos mass spectrometer in positive ion mode. The MS1 scanning range was set to m/z 300–1400 with a resolution of 60,000. The auto gain control was set to 100,000 and HCD with a normalized collision energy of 35% was used for fragmentation.

Traditional TMT labeling experiments utilize MS^2^ fragment ions for quantitative analysis. The “TopSpeed” algorithm was employed for data-dependent scanning, and MS/MS data was collected at a resolution of 50,000. MS/MS data containing all peptide information were analyzed qualitatively and quantitatively by the software Protein Discover (Thermo Fisher Scientific, version 2.4, Waltham, MA, USA) based on the Sequest HT algorithm. Set related parameters are as follows: Protein Database: Rattus norvegicus (Download from UniProt); Enzyme Name: Trypsin; Max Missed Cleavage Sites: 2; Quantification Methods: TMT 10 plex-TMT-126, TMT-128N and TMT-130C; Precursor Mass Tolerances: 10 ppm; Fragment Mass Tolerances: 0.02 Da, unique peptide ≥1. The Percolator algorithm was used to calculate the false discovery rate (FDR) at the peptide level, and the acceptance limit was set at 1% FDR. DAPs between different groups were identified according to a fold change >1.5 or <0.667 and a *p*-value < 0.05.

#### 4.6.5. Bioinformatics Annotation

The DAPs were subjected to bioinformatics annotation, including GO annotation using the database available at http://www.geneontology.org (accessed on 1 December 2022). KEGG pathway enrichment analysis using the database available at http://www.genome.jp/kegg/ (accessed on 1 December 2022). The DAPs were searched against the STRING database version 11.0 (http://www.string-db.org/) (accessed on 5 January 2023) for PPI.

### 4.7. Western Blotting Validation

The frozen rat joint samples were ground into a powder using liquid nitrogen. RIPA lysis buffer (Beyotime, Catalog number: P0013, Shanghai, China) was added, and the mixture was lysed on ice for 30 min. The mixture was then centrifuged at 17,000× *g* for 25 min, and the supernatant protein concentration was determined using a BCA kit (Beyotime, Catalog number: P0012, Shanghai, China). The 5 × protein loading buffer (Beyotime, Catalog number: P0015, Shanghai, China) was added to these samples, and then they were boiled at 98 °C for 10 min. The protein samples were separated using 10% sodium dodecyl sulfate-polyacrylamide gel electrophoresis gel (Beyotime, Catalog number: P0012A, Shanghai, China). The separated proteins were then transferred onto a polyvinylidene fluoride (PVDF) membrane. Next, the membranes were blocked with a blocking buffer (Beyotime, Catalog number: P0023B, Shanghai, China) for 15 min and incubated with primary antibody C1s (ABclonal, Catalog number: A6878, Wuhan, China), C3 (ABclonal, Catalog number: A22265, Wuhan, China), Kng1 (ABclonal, Catalog number: A11638, Wuhan, China), GAPDH (ABclonal, Catalog number:AC001, Wuhan, China) and β-actin (ABclonal, Catalog number:AC004, Wuhan, China) overnight at 4 °C. The dilution ratio of the primary antibodies is always 1:1000. After washing 6 times (5 min each time) using TBST, the membranes were incubated with the Goat Anti-Rabbit IgG antibody (ABclonal, Catalog number: AS029, Wuhan, China) and the Goat Anti-Mouse IgG antibody (ABclonal, Catalog number: AS003, Wuhan, China) for 90 min. The optical density value of the target zone was analyzed using the Image J software (version 1.53r) processing system after using the ECL solution (Beyotime, Catalog number: P0018AS, Shanghai, China).

### 4.8. Molecular Docking

The 3D structures of kininogen1 were predicted using the iterative threading assembly refinement (I-TASSER) server [43], an online automated protein structure prediction tool. To identify the binding cavity, the SiteMap tool was employed, which facilitates the discovery, visualization, and evaluation of protein binding sites [44]. The receptor was preprocessed using the PrepWiz module in the Schrödinger-2021 software package Maestro 10.2 (Complement C3, PDB ID: 1QQF, Complement C1s subcomponent, PDB ID: 8GMN) and generated low-energy conformations of all small molecule compounds via the LigPrep module with the OPLS3 force field. Epik was used to set ionization parameters, and ionization states were assigned at pH 7.0 ± 2.0. Subsequently, the compounds were docked to the protein binding site using the Glide docking program. All the drawings were made in Chimera and annotations were added for some key interactions.

### 4.9. Statistical Methods

Statistical significance between groups was determined using student’s *t*-test. The data are presented as mean ± SD, and *p* < 0.05 was considered significant.

## 5. Conclusions

In this study, TMT proteomics technology was used to clarify the mechanism of amygdalin in treating RA through enrichment analyses of DAPs. The results showed that amygdalin plays an anti-RA role by regulating the complement and coagulation cascade signaling pathways by inhibiting key proteins such as C1s, C3, and Kng1. These findings shed light on the potential of amygdalin as an anti-RA drug and provide insights into its specific mechanism of action.

## Figures and Tables

**Table 1:** qPCR primer sequences.

Primer Name	Forward Primer	Reverse Primer
IL-1β	AGAACATAAGCCAACAAGTGGT	ACAGGTATAGATTCTTCCCCTT
TNF-α	CCTCTTCTCATTCCTGCTCGT	TCCTCCTTGTTGGGACCGAT
β-actin	CGGGACCTGACAGACTACCTC	AAGTCTAGGGCAACATAGCAC

## Data Availability

The authors do not have permission to share data.

## Supplementary Materials

The following supporting information can be downloaded at: https://www.mdpi.com/article/10.3390/molecules28207126/s1. Table S1. DAPs in the heatmap.
