# Supplementary material for: TMT-Based Proteomics Reveal the Mechanism of Action of Amygdalin against Rheumatoid Arthritis in a Rat Model through Regulation of Complement and Coagulation Cascades
Source: Molecules. 2023 Oct 17;28(20):7126. doi: 10.3390/molecules28207126 (PMC10609517; doi:10.3390/molecules28207126)
Supplement: Supplementary file 1 [file molecules-28-07126-s001.zip › molecules-2619387-supplementary.pdf]

Table S1. DAPs in the heatmap.

Protein ID	Gene name	Protein name	Fold change	
			Model/Control	Amygdalin/Model
P07151	B2m	Beta-2-microglobulin	2.33	0.49
P03957	Mmp3	Stromelysin-1	3.77	0.50
O88766	Mmp8	Neutrophil collagenase	3.17	0.60
P48199	Crp	C-reactive protein	4.87	0.30
P01026	C3	Complement C3	2.75	0.56
P08650	C5	Complement C5	1.80	0.64
Q811M5	C6	Complement component	1.50	0.65
Q62930	C9	Complement component C9	2.18	0.49
P08934	Kng1	Kininogen-1	1.35	0.66
Q63514	C4bpa	C4b-binding protein alpha chain	2.12	0.63
Q63515	C4bpb	C4b-binding protein beta chain	2.97	0.49
Q6P6T1	C1s	Complement C1s subcomponent	2.58	0.66
P50115	S100a8	Protein S100-A8	5.11	0.43
P50116	S100a9	Protein S100-A9	3.43	0.53
P20411	Fcer1g	High affinity immunoglobulin epsilon receptor-γ	1.96	0.52
P97636	Il18	Interleukin-18	2.43	0.51
Q06518	Nos2	Nitric oxide synthase, inducible	2.99	0.58
P43527	Caspase-1	Caspase-1	2.58	0.63
P30152	Lcn2	Neutrophil gelatinase-associated lipocalin	3.75	0.60
Q63313	Lbp	Lipopolysaccharide-binding protein	3.68	0.63
P02764	Orm1	Alpha-1-acid glycoprotein	4.72	0.23
Q63341	Mmp12	Macrophage metalloelastase	1.81	0.60
Q63538	Mapk12	Mitogen-activated protein kinase 12	0.50	2.07
P09006	Serpina3n	Serine protease inhibitor A3N	3.04	0.35
P17475	Serpina1	Alpha-1-antiproteinase	1.29	0.66
Q64268	Serpind1	Heparin cofactor 2	0.24	2.23
Q4G075	Serpinb1a	Leukocyte elastase inhibitor A	2.98	0.46
Q6P734	Serping1	Plasma protease C1 inhibitor	2.43	0.51
P20059	Hpx	Hemopexin	2.92	0.40
P16296	F9	Coagulation factor IX	1.14	0.59
P12346	TF	Serotransferrin	1.58	0.57
P06866	Hp	Haptoglobin	1.63	0.50
P20760	Igg-2a	Ig gamma-2A chain C region	0.77	1.62
P02680	Fgg	Fibrinogen gamma chain	0.47	1.89
P14480	Fgb	Fibrinogen beta chain	0.47	1.71
